# Pulmonary Complications of Gastric Fluid and Bile Salts Aspiration, an Experimental Study in Rat

**Published:** 2013-06

**Authors:** Mitra Samareh Fekri, Hamid Reza Poursalehi, Hamid Najafipour, Shahriar Dabiri, Mostafa Shokoohi, Ali Siahposht Khacheki, Nader Shahrokhi, Reza Malekpour Afshar, Mohammad Reza Lashkarizadeh

**Affiliations:** 1 Physiology Research Center, Kerman University of Medical Sciences, Kerman, Iran; 2 Kerman Research Center in Modeling for Health (RCMH), Kerman University of Medical Sciences, Kerman, Iran

**Keywords:** Bile salts, Gastric fluid, Gastroesophageal reflux, Pulmonary fibrosis, Pulmonary inflammation

## Abstract

***Objective(s):*** Gastroesophageal Reflux Disease (GERD) is one of the most common digestive disorders that frequently lead to pulmonary complications due to gastric fluid aspiration. In the present experimental study, chronic aspiration of gastric fluid, its components and bile salts in rat lung was performed to find out the main factor(s) causing pulmonary complications of gastric fluid aspiration.

***Materials and Methods:*** Forty eight male rats weighted 250-300 g were selected in six groups. After anesthesia and tracheal cannulation, the animals received 0.5 ml/kg normal saline, 0.5 ml/kg of whole gastric fluid, 0.5 ml/kg pepsin (2.5 µg/ml), 0.5 ml/kg hydrochloric acid (pH=1.5) or 0.5 ml/kg bile salts (2.5 µg/ml) by injection into their trachea and lungs. In sham group nothing was injected.

***Results:*** Parenchymal and airways inflammation and fibrosis of bronchi, bronchioles and parenchyma were significantly more in the test groups compared to saline and sham groups (*P*<0.001); also inflammation in pepsin and bile salts groups (histopathology scores: 2.87±0.35 and 3.0±0.0 for bronchial, 2.87±0.35 and 2.87±0.35 for bronchioles, 2.87±0.35 and 2.87±0.35 for parenchymal inflammation) were more than hydrochloric acid and gastric fluid groups (1.75±0.46 and 2.5±0.53 for bronchial, 2.0±0.0 and 2.0±0.0 for bronchioles, 2.0±0.0 and 2.0±0.0 for parenchymal inflammation) (*P*<0.05). The same results were found for fibrosis, so that the fibrosis in pepsin and bile salts groups were more than hydrochloric acid and gastric fluid groups (*P*<0.05).

***Conclusion*** *:* The present results suggested that pulmonary complications causing from bile salts and pepsin might be more than gastric juice and hydrochloric acid.

## Introduction

Gastroesophageal disease (GERD) is one of the most common digestive disorders and according to the previous studies 15% of individuals at least once a week and 7% every day experience the symptoms of heartburn and regurgitation. GERD is defined as returning of stomach or bowel contents into the esophagus due to gastric expansion and pressure increase as well as the defect of lower esophageal sphincter (1).

External esophageal symptoms of GERD are produced due to reflux of gastric contents to pharynx and larynx, broncho-pulmonary tree, nose and mouth. This disease may cause chronic coughing, laryngitis, pharyngitis, sinusitis, otitis, chronic sound complications, dental decays and sometimes night suffocation. Repetitive lung aspirations may cause asthma, pulmonary fibrosis, chronic obstructive pulmonary disease (COPD) and pneumonia (1, 2).

GERD associated with regurgitation and repetitive aspiration into superior and inferior airways has been mentioned as a reason for pulmonary diseases like asthma, laryngitis, chronic cough, recurrent pneumonia, voice harshening, pharyngitis and oral diseases (3).

Idiopathic pulmonary fibrosis is a fatal progressive disease with an unknown etiology in which high prevalence of esophagus exposure to acid has observed and this has mentioned as an involved factor in the pathogenesis of this disease (4). Chronic aspiration of gastric fluid into the transplanted lung of rats, caused monocytes infiltration into the bronchioles as well as fibrosis and loss of airways normal structure similar to bronchiolitis obliterans (5). Granulomatous interstitial pneumonitis has reported as well as prominent production of multinucleated giant cells in lung tissue after chronic aspiration of gastric fluid in the rat lung, also chronic aspiration of pH-neutralized gastric fluid is showen similar pathology of chronic acid aspiration (6). An investigation has studied the effect of chronic aspiration of gastric fluid in the inflammatory response of rat lung. Histological studies of lung samples showed fibrosis profile, giant cells infiltration, lymphocytic bronchiolitis, bronchiolitis obliterans as well as increase in inflammatory interleukins in lung lavage fluid (7).

Repetitive acid aspiration in rodents’ lung has caused an increase in TGF-β1 in broncho-alveolar lavage (BAL) and an increase in incidence of collagen III/IV and fibronectin in lung tissue. These may show profibrotic mechanism and pulmonary fibrosis due to acid aspiration (8). Aspiration due to duodenal-gastroesophagial reflux is prevalent after lung transplantation and this is recognized with an incidence of bronchiolitis obliterans along with fibrosis and inflammatory cell infiltration around small airways (9). In asthmatic patients, GERD has been known as an important factor in asthma attacks (10) and oxidative stress intensification (11). It has shown that disturbance in swallowing in COPD patients are along with exacerbation of acute disease attacks (12).

One study about the effect of gastric contents reflux in patients with lung allograft (with or without chronic graft reject) has reported the presence of pepsin in the content of bronchial lavage fluid of all patients and bile salts in 50% of them. Bile salts were observed in the BAL of 70% of patients with bronchiolitis obliterans in comparison to 31% of patients who had stable condition (13). A research has proved that the addition of bile acids to the type II pneumocytes causes intracellular calcium accumulation and is probably the cause of pneumonitis resulted from aspiration (14). As gastric fluid contains hydrochloric acid, food particles, pepsin, mucus and other enzymes and the precise effect of chronic aspiration of each component on pulmonary damage has not yet been identified and in many cases bile salts enter stomach via duodenum and are aspirated accompanying gastric fluid as well, in the present experimental study, chronic aspiration of gastric fluid, its components and bile salts were performed in rat lung to find out which component is more responsible for pulmonary complications of GERD; also to find out whether treatment of patients with pulmonary complications of GERD should be limited to traditional anti-acid therapy, or alternative therapeutic methods are necessary. 

## Materials and Methods

This study was performed on Albino N-more rats weighing 250-300 g. The number of animals in each group was determined based on similar previous studies (6, 7, 15). Animals were housed in cages of 4 rats in the animal house of Kerman Faculty of Medicine at the temperature of 20-22°C and 12h dark-light cycle. They had free access to food and water. All procedure and animal care were approved by the ethics committee of Kerman University of Medical Sciences (Permit No: KA 89/44 (. Animals were randomly allocated to 6 groups (n=8 each) and after undergoing anesthesia and tracheal cannulation (see below; chronic aspiration section for details) the following substances were injected into their trachea:

1. Sham group: anesthetized and cannulated with no substance injection.
2. Saline group: 0.5 ml/kg normal saline injection
3. Gastric fluid group: 0.5 ml/kg whole gastric fluid injection
4. Pepsin group: 0.5 ml/kg pepsin at concentration of 2.5 µg/ml injection
5. Hydrochloric acid group: 0.5 ml/kg hydrochloric acid (pH =1.5) injection
6. Bile salts group: 0.5 ml/kg bile salts at concentration of 2.5 µg/ml injection

The above amounts injected were determined based on previous studies which in rats gastric fluid had been collected for 15 min, and the concentrations of mentioned substances measured (6, 7). Since in normal conditions there is no bile salt in gastric fluid, the amount of injected bile salts was selected based on the concentration of pepsin in gastric fluid and in order the results to be compared.

***Gastric fluid collection***

After being fasted for 12 hr, animals were anesthetized by intra-peritoneal injection of sodium thiopental. Abdomen was opened and after ligation of the end of esophagus with a suture, duodenum was incised few cm distal to pyloric sphincter. Then a catheter connecting to a syringe was placed into the stomach via duodenum in which 4 ml distilled water was injected into the stomach. After 15 min gastric contents was withdrawn and transferred into 1-ml Eppendorf tubes to be kept on -80°C until the time of the experiments (6, 7). 

***Chronic aspiration***

 After anesthesia with ether, the animal was placed on the back on a 45° angle inclined plane. The animal’s head was bent from the upper part of the plane and by using a cold light source animal’s throat was shined. The mouth was opened and by observing inhaling and exhaling the tracheal opening was detected and tracheal cannulation was performed (6, 7, 15). Previously prepared materials kept in 0.5 ml/kg volumes in 1-ml syringes were slowly injected into the catheter. Then the animal was placed on the right side with maintaining the head higher than the body for 5 min allowing the injected fluid to enter the right lung while preventing the inflammation of the left lung and consequently early death. During all this time the animal was maintained in anesthetized condition by using an ether mask and after regaining consciousness it was controlled for one hr and then transferred to the cage.

Injections were performed under short-term anesthesia at 8-10AM twice a week for a duration of 8 weeks. One week after the last injection, the animals were anesthetized with intra-peritoneal injection of 50 mg/kg sodium thiopental. Then by making an incision in thorax area, lung was isolated along with trachea and fixed in 10% formalin solution. After coding the specimens, they were sent to the laboratory for pathological study. 

***Histopathology study***

The samples were kept in 10% formalin solution for 24 hr and then paraffin blocks were prepared. Tissue passage stages were performed by tissue processor instrument (LEICA, Germany). From each lung, ten 5-µ slices were prepared by microtome and half of them were stained by H&E staining technique for studying inflammation indices, and the other half were stained by Masson's trichrome for studying fibrosis. The pathologist was blind to the animal’s group. For each specimen a report form with special code was filled out. 

The pathologist evaluated samples for: a) the rate of inflammatory infiltration (16-18) tissue fibrosis (19), the presence of specific types of bronchiolitis including bronchiolitis obliterans and granulomatous bronchiolitis (22) as follows:

***a) Inflammatory infiltration (0-4 grade):***

0: No inflammation

1: Occasional cuffing with inflammatory cells

2: Most bronchi or vessels surrounded by a thin layer (1-5 cells thick) of inflammatory cells

3: Most bronchi or vessels were surrounded by a thick layer (more than 5 cells thick) of inflammatory cells

4: Total lung inflammation: perivascular inflammation and peribronchial inflammation (16-18)

***b) Criteria for grading lung fibrosis grade of histological features fibrosis***

0: Normal lung

1: Minimal fibrous thickening of alveolar or bronchiolar walls

2,3: Moderate fibrous thickening of walls without obvious damage to the lung architecture

4,5: Increased fibrosis with definite damage to the lung structure and formation of fibrous bands or small fibrous masses

6, 7: Severe distortion of structure and large fibrous areas;”honeycomb lung”is placed in this category

8: Total fibrous obliteration of the field (19)

***c) The presence of specific types of bronchiolitis***

- Bronchiolitis Obliterans (obstruction of bronchioles by organized exudate)

- Granulomatous bronchiolitis (the presence of epithelioid granulomas with multi-nucleated giant cells (22)

***Statistical analysis***

Data are presented as mean±SD. Non-parametric test of Kruskal Wallis was used to the difference of the indices among groups followed by Tukey HSD for intergroup comparison. Data analysis was done by SPSS15 and *P*<0.05 was considered as significant level.

## Results

***Parenchyma and bronchioles inflammation***

In regard to the Parenchyma and bronchioles inflammation, test groups showed no significant difference with each other, but they showed significant increase when compared to sham and normal saline groups (*P*<0.001). Pepsin and bile salts groups showed a significant increase (*P*<0.05) in these two variables compared to hydrochloric acid and gastric fluid groups (Table 1) (Figures 2-5 versus 1).

***Bronchitis***

The rate of bronchitis was significantly different among test groups (*P*<0.001) in a way that it was significantly more in hydrochloric acid, gastric fluid, pepsin and bile salts groups compared to sham and saline groups (*P*<0.05). The bile salts group showed a significant increase in bronchitis in comparison to both hydrochloric acid and gastric fluid (*P*<0.05), but pepsin group showed a significant increase in bronchitis (*P*<0.05) only in comparison to hydrochloric acid group (Table 1). 

**Table 1:** The effect of aspiration of gastric fluid and its components on inflammation of bronchi, bronchioles and parenchyma in the studied groups (mean ±SD)

Groups	Bronchial inflammation	Bronchioles inflammation	Parenchyma inflammation
Sham	0.0±0.0	0.0±0.0	0.0±0.0
Normal saline	0.0±0.0	0.125±0.35	0.125±0.35
Hydrochloric acid	1.75±0.46^*^	2.0±0.0^*^	2.0±0.0^ *^
Gastric fluid	2.5±0.53^*,¥^	2.0±0.0^ *^	2.0±0.0^ *^
Pepsin	2.87±0.35^ *,¥ ^	2.875±0.35^ *, £^	2.875±0.35^ *, £^
	3.0±0.0^*,£^	2.875±0.35^ *, £^	2.875±0.35^ *, £^
*P*- value	<0.001	<0.001	<0.001

*: *P*<0.05 in comparison to sham and normal saline groups

£: *P*<0.05 in comparison to hydrochloric acid and gastric fluid groups

¥:*P*<0.05 in comparison to hydrochloric acid group

**Figure 1:**
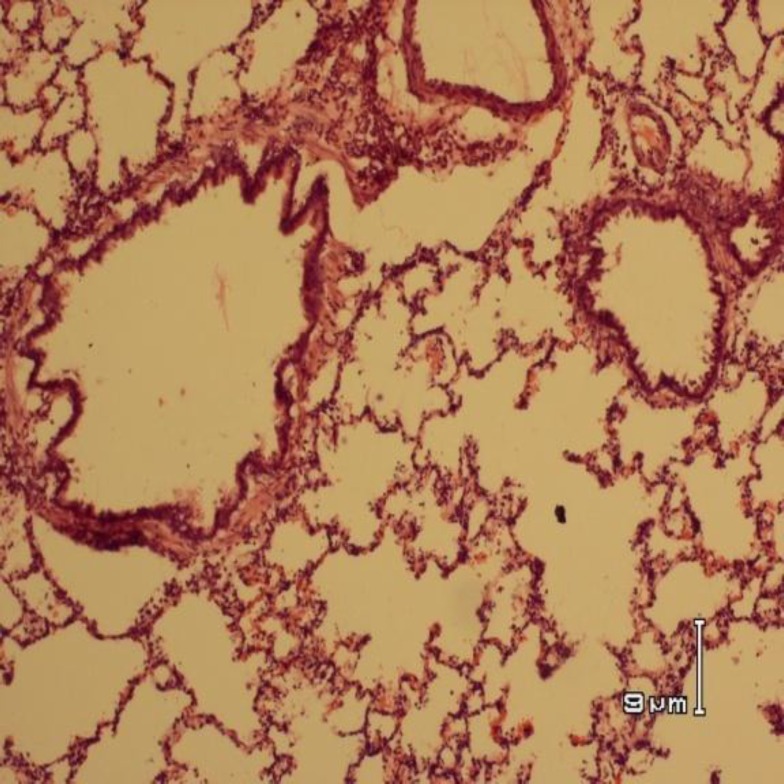
Histopathology of the lung of an animal from sham group showing terminal and respiratory bronchioles with normal pulmonary epithelium and muscular wall, and also normal alveolar spaces , wall and capillaries (H&E staining, * 40 magnification)

**Figure 2:**
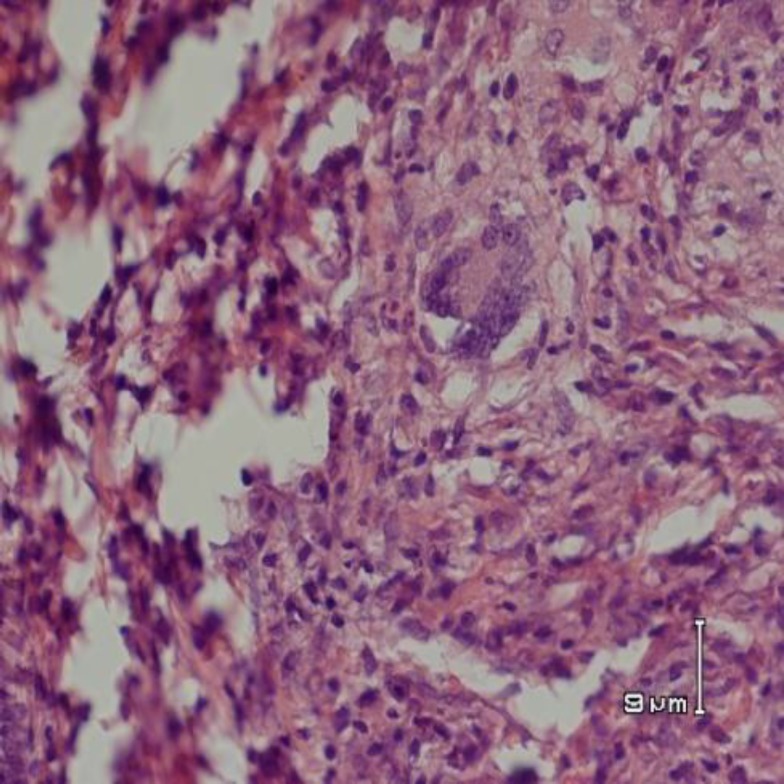
Histopathology of the lung of an animal from pepsin group showing non caseating granulomatous composed of lymphohistiocytic collection and langhan,s type giant cells. (In the center of the picture) (H&E staining, * 200 magnification)

**Figure 3:**
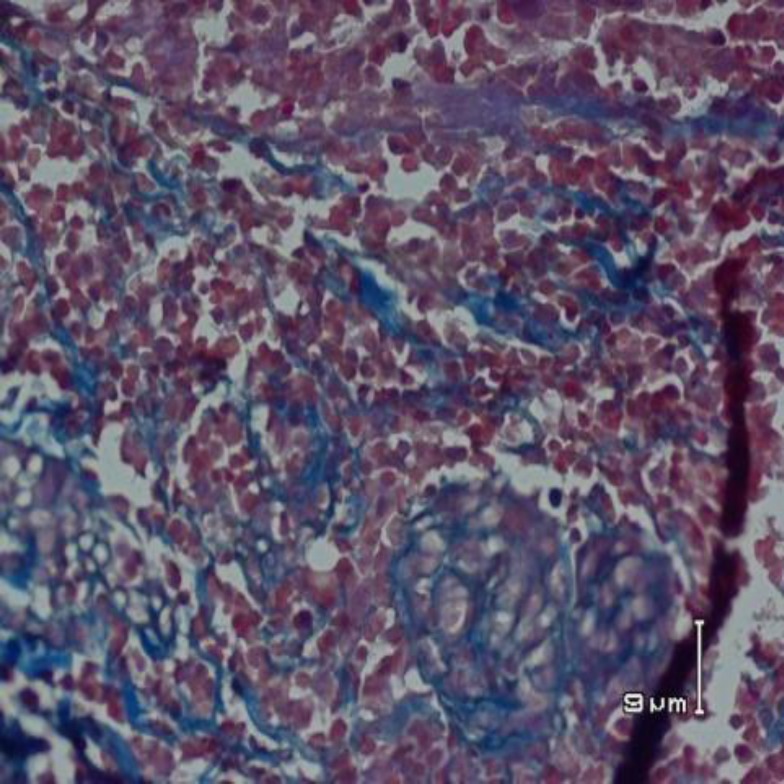
Histopathological changes of bronchus of pepsin group showing severe hyperemia and mononuclear cell infiltration with the destruction of connective tissue framework and its cartilage. (Mason trichrom staining, * 400 magnification)

**Figure 4:**
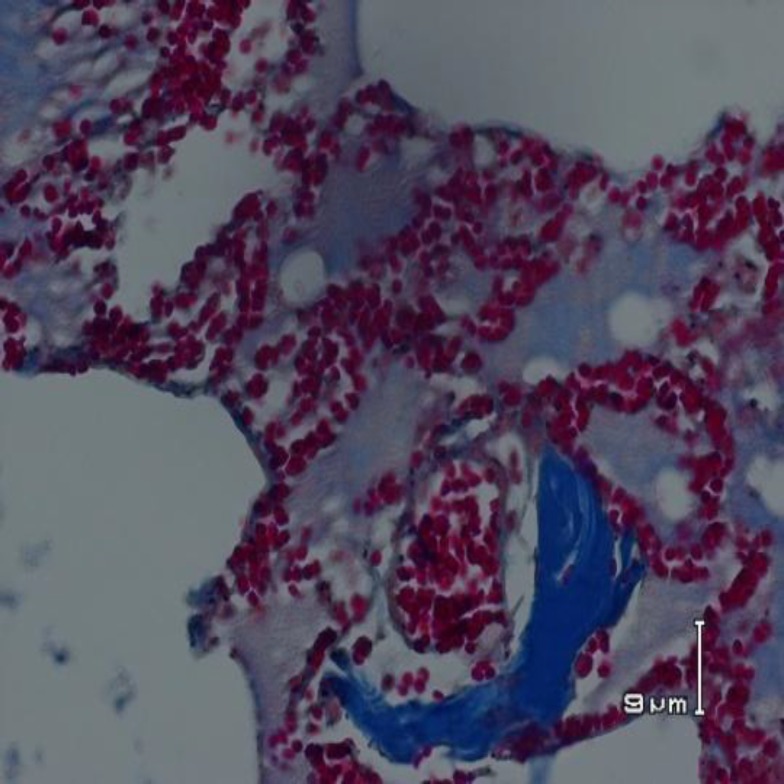
Histopathological changes of an animal from bile salts group showing aggregation of collagen fibers in alveoli are seen focally (Mason trichrom staining, * 200 magnifications)

**Figure 5:**
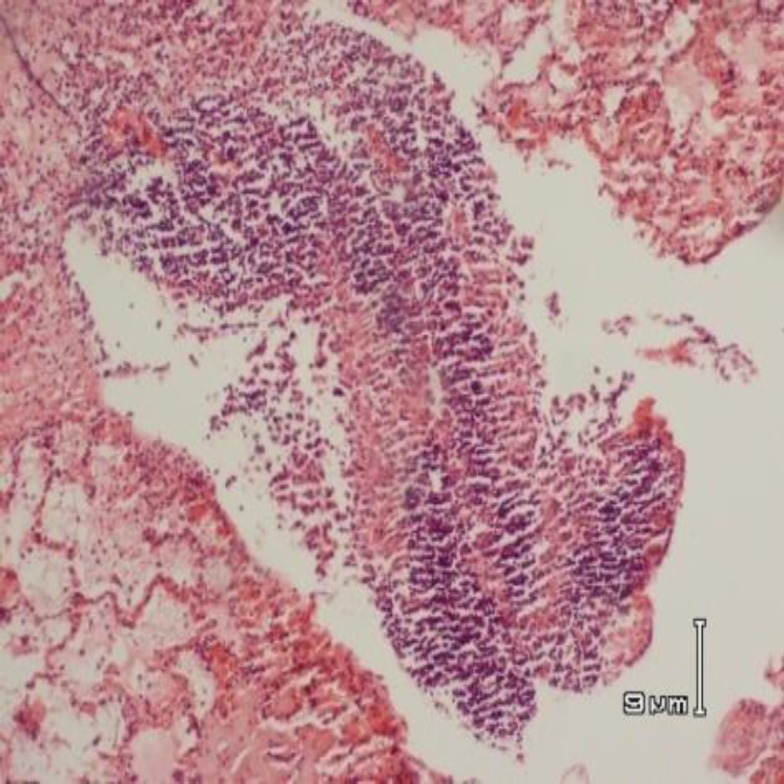
Histopathological changes of an animal from the bile salts group showing ulceration of the mucosa in terminal and respiratory bronchioles along with the fibrin exudate formation and severe exfoliation of surface epithelial cells in respiratory bronchioles. Severe mononuclear cell infiltration is seen in the lamina propria (H&E staining, * 100 magnification)

***Parenchyma and bronchioles fibrosis***

As it is presented in Table 2, these variables were significantly different among test groups (*P*<0.001). Moreover, hydrochloric acid, gastric fluid, pepsin and bile salts had significantly higher grades of fibrosis in comparison to sham and saline groups (*P*<0.05). 

Pepsin and bile salts groups showed significant increases in parenchyma and bronchioles fibrosis compared to hydrochloric acid and gastric fluid (*P*<0.05). These two variables in the bile salts group were significantly higher than the corresponding values in pepsin group (*P*<0.05) (Figures 4٬5 versus 2٬3).

**Table 2:** The effect of aspiration of gastric fluid, its components and bile salts on fibrosis of bronchi, bronchioles and parenchyma in the studied groups (mean ±SD)

Groups	Bronchial fibrosis	Bronchioles fibrosis	Parenchyma fibrosis
Sham	0.0±0.0	0.0±0.0	0.0±0.0
Normal saline	0.0±0.0	0.0±0.0	0.0±0.0
Hydrochloric acid	1.62±0.51^*^	1.0±0.0^ *^	1.0±0.0^ *^
Gastric fluid	2.12±0.35^*^	1.0±0.0^ *^	1.0±0.0^ *^
Pepsin	2.5±0.53^ *, ¥^	1.62±0.51^ *, £^	1.62±0.51^ *, £^
Bile salts	2.87±0.35^*,£^	2.0±0.0^ *, £, €^	2.0±0.0^ *, £, €^
*P- value*	<0.001	<0.001	<0.001

*: P<0.05 in comparison to sham and normal saline groups;

£: *P*<0.05 in comparison to hydrochloric acid and gastric fluid groups

¥: *P*<0.05 in comparison to hydrochloric acid group;

€: *P*<0.05 in comparison to pepsin group

**Table 3:** Granuloum and bronchiolitis obliterans grades in the studied groups (mean ±SD)

Groups	Granuloum	Bronchiolitis obliterans
Sham	0.0±0.0	0.0±0.0
Normal saline	0.0±0.0	0.0±0.0
Hydrochloric acid	0.125±0.35	0.0±0.0
Gastric fluid	0.0±0.0	0.125±0.35
Pepsin	0.25±0.46	0.125±0.35
Bile salts	0.0±0.0	0.37±0.51
*P*- value	0.196	0.10

***Bronchial fibrosis***

Hydrochloric acid, gastric fluid, pepsin and bile salts groups in comparison to sham and saline groups had higher rates of bronchial fibrosis (*P*<0.05). Bile salts group in comparison to hydrochloric acid and gastric fluid groups showed significant increases in bronchial fibrosis (*P*<0.05), while pepsin group only in comparison to the hydrochloric acid group had a significant increase in this item (*P*<0.05, Table2).

As it is seen in Table 3, there is no significant difference among test groups in regard to the rate of granuloum and bronchiolitis obliterans.

## Discussion

In the present experimental study, the effects of chronic aspiration of gastric fluid, pepsin, hydrochloric acid and bile salts were investigated on rat lung airways and parenchyma. Based on the results, intensity of pulmonary damage resulting from aspiration of bile salts and pepsin was significantly more than that of hydrochloric acid and whole gastric fluid. The relationship between chronic aspiration and pulmonary idiopathic fibrosis is well founded. According to them, chronic aspiration of gastric contents into the airways causes tissue fibrotic response after pulmonary damage (20) and this is in line with the results of the present study in regard to inflammation and fibrosis due to chronic aspiration. However, it has not determined that which gastric content causes more pulmonary complications while the present study cleared that damages resulting from bile salts and pepsin were more severe compared to gastric fluid and hydrochloric acid. Another study also claimed the association between GERD and idiopathic pulmonary fibrosis. In that study, from seventeen patients whose lung biopsy showed idiopathic pulmonary fibrosis, sixteen patients had abnormal acid exposure in the distal or proximal esophagus in comparison to the control group (4). It just studied the effect of gastric acid, but in the present study, the effects of all gastric contents have been investigated. One study has shown gastric fluid aspiration caused inflammatory reactions in both superior and inferior airways (21) which is in agreement with our study, but in that study pulmonary complications of gastric fluid components was not studied. A research has reported higher prevalence of bronchiolitis obliterans in non-acid reflux group of lung transplant patients compared to acid reflux group (22). In the present study, inflammation and fibrosis around bronchioles resulted from pepsin and bile salts aspiration were more than those due to acid aspiration but no bronchiolitis obliterans was observed. This difference between the two studies may be due to the type of study (animal vs. human). Meanwhile, the mentioned study was performed on transplanted lung which is exposed to various immune mechanisms that can affect the results. An investigation has proved the effects of chronic aspiration of gastric fluid in lung transplant rats and observed higher incidence of bronchiolitis obliterans in animals with gastric fluid aspiration compared with the control group (5) which differs from the results of the present study. The difference may be attributed to the immune system deficiency in lung transplant animals that had made them more vulnerable to damaging factors. The same thing may be true for another study of lung transplant patients in which 70% of patients with bronchiolitis obliterans (compared to 31% stable patients) had bile salts in bronchoalveolar lavage fluid (13). It has reported aspiration of acid and pH-neutralized gastric fluid caused significantly higher rates of vascular parenchyma inflammation and production of giant cells and granolumes in the experimental group compared to the control group (6). Again in the mentioned study, only the effect of aspiration acid from gastric fluid was investigated and did not show which of the gastric fluid components were responsible for observed complications. It has shown that amount of bile acids in sputum was higher in asthma patients with clinical symptoms of GERD compared to those without GERD symptoms (23). The results of the present study in which chronic aspiration of bile salts caused higher rates of inflammation and fibrosis in parenchyma and airways confirm that bile salts may be a main determinant of problems in patients with GERD symptoms. Bile salts cause changes in the permeability of cell membrane to cations and this has been confirmed in type II pneumocyte cells and disturbance in pulmonary function in lung transplant patients because of the fact that the bile salt aspiration is mediated by IL-8 production and alveolar neutrophilia (24).

The effect of repetitive microaspiration in animal model has shown that histopathologic changes in lung were as prebronchial neutrophil infiltration and Goblet cell hyperplasia, increased in blood vessel and smooth muscle thickness around the airways (25). These findings are similar to the results of the present study.

The effect of chronic aspiration of gastric fluid in a rat model of lung transplantation was studied and it was reported monocyte infiltration, fibrosis and loss of normal alveolar anatomy (15). That study has also the shortcoming of not clearing which gastric components are more responsible for the observed pulmonary complications. As it is presented in tables 1 and 2, bile salts have caused the highest rates of pulmonary complications. Although gastric fluid contains both acid and pepsin but did not cause more complications compared with each alone. This finding implies that there are probably some protective substances in the gastric fluid, maybe mucus, but these assumptions require more studies to prove.

No pathologic complication in sham and normal saline groups show that none of the manipulations of tracheal cannulation or fluid aspiration are the cause of pathologic changes observed in the lung. Therefore, pulmonary complications of GERD are related to the components of gastric fluid, or to bile salts that have entered from the duodenum into the stomach and then aspirated to the airways.

## Conclusion

This experimental study showed the association of chronic gastric fluid aspiration or its components with inflammation and fibrosis in lung parenchyma and airways. Therefore, it is suggested that all patients with advanced pulmonary disease to be studied for GERD. Control of reflux in the primary stages of disease may improve lung function or stop the progress of the disease. As pulmonary complications of gastric fluid aspiration are not just related to its acid content, anti-reflux surgeries such as fundoplication should be used in the first step and anti-acid medications and hydrogen pump inhibitors should be used as auxiliary treatments. 

***Study limitations***

Since the results of studies using animal models cannot be generalized to human, more studies are required to find the complications resulting from different components of gastric fluid reflux in human. Another limitation of the present study was the small number of weekly aspirations that may not be a representative of clinical gastroesophageal reflux in which reflux occurs more frequently. However, it is expected that the intensity of complications to increase with more aspirations. We could not increase the number of aspirations per week, because based on the pilot study, more aspirations results in a high rate of animal deaths due to the invasiveness of experiment process (anesthesia, tracheal cannulation and injections). 

## References

[1] Kasper DL, Braunwald E, Fauci AS, Kasper DL, Fauci AS, Longo DL (2008). Diseases of the esophagus in: Harrison's principles of internal medicine.

[2] Richter JE (1996). Typical and atypical presentations of gastro esophageal reflux disease. The role of esophageal testing in diagnosis and management. Gastroenterol. Gastroenterol Clin North Am.

[3] Poelmans J, Tack J (2005). Extra-oesophageal manifestations of gastro esophageal reflux. Gut.

[4] Tobin RW, Pope CE 2nd, Pellegrini CA, Emond MJ, Sillery J, Raghu G (1998). Increased prevalence of gastroesophageal reflux in patients with Idiopathic pulmonary fibrosis. Am J Respir Crit Care Med.

[5] Li B, Hartwig MG, Appel JZ, Bush EL, Balsara KR, Holzknecht ZE (2008). Chronic Aspiration of Gastric fluid Induces the Development of obliterative bronchiolitis in Rat lung Transplants. Am J Transplant.

[6] Downing TE, Sporn TA, Bollinger RR, Davis RD, Parker W, Lin SS (2008). Pulmonary histopathology in an experimental model of chronic aspiration is independent of acidity. Exp Biol Med.

[7] Appel JZ, Lee SM, Hartwig MG, Li B, Hsieh CC, Cantu E (2007). Characterization of the innate immune response to chronic aspiration in a novel rodent model. Respir Res.

[8] Kwan M, XU YD, Raghu G, Khalil N, Vancouver BC, Seattle WA (2007). Acid treatment of normal rat lungs releases transforming growth factor –beta 1 (TGF-beta 1) and increases connective tissue synthesis. Am J Respir Crit Care Med.

[9] D'Ovidio F, Mura M, Tsang M, Waddell TK, Hutcheon MA, Singer LG (2005). Bile acid aspiration and the development of bronchiolitis obliterans after lung Transplantation. J Thorac Cardiovasc Surg.

[10] Harding SM (2005). Gastroesophageal reflux: a potential asthma trigger. Immunol Allergy Clin North Am.

[11] Carpagnano GE, Resta O, Ventura MT, Amoruso AC, Di Gioia G, Giliberti T (2006). Airway inflammation in subjects with gastro-oesophageal reflux and gastro-oesophageal reflux-related asthma. J Intern Med.

[12] Terada K, Muro S, Ohara T, Kudo M, Ogawa E, Hoshino Y (2010). Abnormal swallowing reflex and COPD exacerbations. Chest.

[13] Blondeau K, Mertens V, Vanaudenaerde BA, Verleden GM, Van Raemdonck DE, Sifrim D (2008). Gastro-oesophageal reflux and gastric aspiration in lung transplant patients with or without chronic rejection. Eur Respir J.

[14] Oelberg DG, Downey SA, Flynn MM (1990). Bile salt induced intracellular Ca++ accumulation in type II pneumocytes. Lung.

[15] Hartwig MG, Appel JZ, Li B, Hsieh CC, Yoon YH, Lin SS (2006). Chronic aspiration of gastric fluid accelerates pulmonary allograft dysfunction in a rat model of lung transplantation. J Thoracic Cardiovasc Surg.

[16] Sur S, Wild JS, Choudhury BK, Sur N, Alam R, Klinman DM (1999). Long term prevention of allergic lung inflammation in a mouse model of asthma by CpG Oligodeoxynucleotides. J Immunol.

[17] Kwak YG, Song CH, Yi HK, Hwang PH, Kim JS, Lee KS (2003). Involvement of PTEN in airway hyper responsiveness and inflammation in bronchial asthma. J Clin Invest.

[18] Cho KJ, Seo JM, Shin Y, Yoo MH, Park CS, Lee SH (2010). Blockade of airway inflammation and hyperresponsiveness by inhibition of BLT2, a low-affinity leukotriene B4 receptor. Am J Respir Cell Mol Biol.

[19] Ashcroft T, Simpson JM, Timbrell V (1988). Simple method of estimating severity of pulmonary fibrosis on a numerical scale. J Clin Pathol.

[20] Lee JS, Collard HC, Raghu G, Sweet MP, Hays SR, Campos GM (2010). Dose chronic microaspiration cause idiopathic pulmonary fibrosis?. Am J Med.

[21] Hoyoux C, Forget P, Lambrechts L, Geubelle F (1985). Chronic broncho pulmonary disease and gastroesophageal reflux in children. Pediatr Pulmonol.

[22] King BJ, Iyer H, Leidi AA, Carby MR (2009). Gastroesophageal reflux in bronchiolitis obliterans syndrome: a new perspective. J Heart Lung Transplant.

[23] Perng DW, Chang KT, Su KC, Wu YC, Wu MT, Hsu WH (2007). Exposure of airway epithelium to bile acids associated with gastroesophageal reflux symptoms: a relation to transforming growth factor-beta1 production and fibroblast proliferation. Chest.

[24] DiGiovine B, Lynch JP 3rd, Martinez FJ, Flint A, Whyte RI, Iannettoni MD (1996). Bronchoalveolar lavage neutrophilia is associated with obliterative bronchiolitis after lung transplantation. Role of IL-8.

[25] Oue K, Mukaisho K, Higo T, Araki Y, Nishikawa M, Hattori T (2011). Histological examination of the relationship between respiratory disorders and repetitive microaspiration using a rat gastro-duodenal contents reflux model. Exp Anim.

